# Dream engineering: Simulating worlds through sensory stimulation

**DOI:** 10.1016/j.concog.2020.102955

**Published:** 2020-07-08

**Authors:** Michelle Carr, Adam Haar, Judith Amores, Pedro Lopes, Guillermo Bernal, Tomás Vega, Oscar Rosello, Abhinandan Jain, Pattie Maes

**Affiliations:** a Sleep & Neurophysiology Research Laboratory, Department of Psychiatry, University of Rochester Medical Center, Rochester, NY, USA; b MIT Media Lab, MIT, Boston, MA, USA; c University of Chicago, Chicago, IL, USA

**Keywords:** Dreaming, Human Computer interaction, Haptic devices, Virtual reality, Simulation, Sleep

## Abstract

We explore the application of a wide range of sensory stimulation technologies to the area of sleep and dream engineering. We begin by emphasizing the causal role of the body in dream generation, and describe a circuitry between the sleeping body and the dreaming mind. We suggest that nearly any sensory stimuli has potential for modulating experience in sleep. Considering other areas that might afford tools for engineering sensory content in simulated worlds, we turn to Virtual Reality (VR). We outline a collection of relevant VR technologies, including devices engineered to stimulate haptic, temperature, vestibular, olfactory, and auditory sensations. We believe these technologies, which have been developed for high mobility and low cost, can be translated to the field of dream engineering. We close by discussing possible future directions in this field and the ethics of a world in which targeted dream direction and sleep manipulation are feasible.

## 1. Introduction

Dreaming is often considered a subjective experience generated by the mind and brain, while cut off from the body and the external environment (Brueckner, 1986). In philosophy, this view is referred to as *brain in a vat* consciousness, whereby the brain generates experience even in the absence of physical input or outward control. One of the logical consequences of any *brain in a vat* theory is that bodiless brains attached to reality simulators would still have the same experience we are having right now. This position is juxtaposed by the richly embodied and immersive experiences brought about in dreams, where we feel our dreamt bodies and engage with our imagined environments. Proponents of neurocognitive theories of dreaming suggest that such experiences are akin to *simulations* of the waking world (Foulkes, 1985; Revonsuo, 2000; Tart, 1987; see Nielsen, 2010 for a review). They are created from memory – recent and remote – and come alive through sensorimotor, limbic, and default-mode cortical activity. In Hobson’s theory of protoconsciousness (Hobson, 2009; Hobson, Hong, & Friston, 2014), the REM state is viewed as a virtual reality (VR) generator used by the brain to instantiate world interactions and to build predictions of space and time.

Yet dreamed experience does not occur in isolation from the sleeping body. Dreaming shows one-to-one correspondences – termed isomorphisms – between the experiences of the dreaming body and the ‘real’ sleeping body. Bodily sources of dream imagery include sensory incorporation, muscular twitches, and other isomorphisms between eye movements, respiration, motor circuitry, and heart rate (Dement & Wolpert, 1958). Thus, Windt (2018) proposes that dreams are “phenomenally-functionally embodied simulations” Phenomenal embodiment refers to the subjective experience of being-in-a-world, a key in simulation views of dreaming. Functional embodiment refers to “the causal relationship between phenomenal embodiment on the one hand and sensory inputs and motor outputs on the level of the physical body on the other hand.” In other words, the dreaming brain is not in a vat; it is in a circuit with the sleeping body.

Framing dreams as functionally embodied opens up an avenue to manipulating the dreaming mind: the body as a permeable barrier that can be used to interface with the virtual world of dreams. Parallel work in Human-Computer Interaction (HCI) has utilized the body as an interface to increase the immersion of *virtual* environments by engineering multi-modal devices that can simulate haptic sensations such as touch, temperature, and inertial forces as well as audio-visual or olfactory sensations. We believe these systems are prime candidates to influence dreams on demand and in ambulatory settings. In this review, we present a model of dreaming as being in circuit with the sleeping body and adapt HCI approaches to dream engineering. We discuss a set of technologies to influence sleep for memory enhancement, creativity, emotion regulation and physical rehabilitation. Possible future directions in dream engineering solutions and ethical considerations will be discussed.

### 1.1. Isomorphisms in the dreaming and sleeping body

The discovery of the “rapid, jerky, binocularly symmetrical” eye movements of REM sleep prompted a surge in scientific studies of dreaming, after 74% of REM awakenings were associated with recall of complex, visual and emotion-filled dreams (Aserinsky & Kleitman, 1953). Rapid eye movements thus became the first objective physiological correlate of conscious experience during sleep. It was later suggested that rapid eye movements correspond with visual scanning of the dreamscape—known as the scanning hypothesis (Dement & Kleitman, 1957a, 1957b). Herman et al. (1984) supported this claim, finding that eye movements produced just before waking matched visual descriptions of dream imagery, although other findings are inconsistent (Jacobs, Feldman, & Bender, 1972; Moskowitz & Berger, 1969). In studies of lucid dreaming, where the dreamer is aware that they are dreaming and able to control their dreaming body to some extent, further evidence supports an isomorphism between dreamed and actual eye movements. Researchers (Hearne, 1978; LaBerge, 1980; LaBerge, Nagel, Dement, & Zarcone, 1981) demonstrated that lucid dreamers were able to signal using deliberate eye movements, such as looking to the left and right, which corresponded with left-right eye movements recorded by an electrooculogram (EOG). The body could, with verifiable physiological signals, bridge dream states with the waking world.

Since then, lucid dreaming and other dream phenomena such as somnambulism and REM behavior disorder (RDB) have revealed further correspondences between the sleeping and the dreaming body. For instance, lucid dreamers can induce muscular twitches in the actual forearm through dream movements (LaBerge et al., 1981) and can actively influence respiration, abdominal and thoracic muscle movements (Oudiette et al., 2018). The authors of the latter study suggest that even in nonlucid dreams the content of dream experience may physically manifest in the sleeping body, which may in part explain the erratic respiration, changes in heart rate, and muscular activity exhibited particularly during REM sleep. For example, nightmares are associated with elevated heart rate (Fisher, Byrne, Edwards, & Kahn, 1970) and increased eye movements and respiration rate (‘high anxiety dreams’; Goodenough, Witkin, Koulack, & Cohen, 1975), which suggests the body is physically experiencing a nightmare as long as the mind is. This view has substantial implications for Posttraumatic Stress Disorder (PTSD) where patients re-experience a trauma during nightmares (Van der Kolk, 1994), leading authors to claim that ‘dreams themselves could be traumatic for the dreamer’ (Campbell & Germain, 2016). Here, we expand on the argument for a stronger role of dreaming in sleeping physiology than has previously been acknowledged and, subsequently, explore the functional implications of this claim.

### 1.2. Sources of dreaming

#### 1.2.1. Bottom-up bodily sensations

During REM sleep, extensive sensorimotor cortical activity seems to underlie the vivid embodied imagery of dreaming. Siclari et al. (2017) showed that dream content corresponds with activation in appropriate perceptual areas of the brain; for instance perception of faces in dreams is predicted by increased activation in the fusiform face area, similar to in wakefulness. And, functional magnetic resonance imaging (fMRI) of lucid dreams showed that dreamed hand movements correspond to activity in the contralateral sensorimotor cortex (Dresler et al., 2011), and manifest as muscular twitches in the forearm (LaBerge et al., 1981). The activation-synthesis hypothesis of dreaming proposed that sensorimotor cortical activity is generated by brainstem signals during REM sleep and that muscular twitches are simply a by-product of incomplete motor blockade (Hobson & McCarley, 1997), thus reducing the dreaming circuitry to a *brain in a vat* simulation. However, Blumberg (2010) claims that sensory feedback from muscular twitches produces substantial activation of the somatosensory cortex during REM sleep. Key characteristics include that twitches occur in discrete, repetitive patterns throughout the body against a backdrop of muscle atonia (Robinson, Blumberg, Lane, & Kreber, 2000) and cause amplified and repetitive patterns of activation in the somatosensory cortex. Critically, this feedback completes the circuitry between the dreaming brain and the sleeping body and suggests twitches may be an active bodily source of dream generation.

Perhaps the most common example of dreaming being influenced by muscular twitches occurs during sleep onset when the experience of hypnic jerks can be associated with vivid imagery of falling (Nielsen & Zadra, 2005); a similar interpretation could be provided for “exploding head syndrome” in that twitches of the middle ear muscle (the “hearing” correlate to eye movements) might suddenly be amplified as the body falls into quiescence. In the case of RBD, violent dreams seem to be enacted in often repetitive violent movements, such as kicking or punching (Valli et al., 2015). Blumberg and Plumeau (2016) suggest the loss of muscle atonia in RBD leads to excessive twitching, and the resulting sensory feedback gives rise to vivid and violent dreams. It is otherwise difficult to determine why suddenly, at the onset of RBD, patients would develop such violent dreams. Finally, a recent study showed that using transcranial direct current stimulation to inhibit sensorimotor cortex activity specifically decreased the presence of repetitive actions in REM sleep dreams (Noreika et al., 2020). The authors interpret these findings as evidence that the sensorimotor cortex is causal in the generation of dream movement. However, the specificity of inhibition to repetitive, but not other movement types, could reflect inhibited sensory feedback from repetitive twitches.

Beyond muscular twitches, other internal body states may also play a role in dreaming cognition. For instance, changes in breathing, heartrate, metabolism or circadian rhythm all correspond with changes in the quality of subjective experience in waking (see Critchley & Garfinkel, 2018 for a review). Recent evidence that physiological signals influence dream content is shown in Perogamvros et al. (2019), who found increased heartbeat-evoked potentials during REM sleep in nightmare sufferers. The heartbeat-evoked potential represents cortical response to the heartbeat and is an index of interoception, i.e. the sense of internal body states. Other general support includes evidence that bodily states such as fever (Schredl & Erlacher, 2020), hunger and thirst (O’Nell, 1965) have been associated with changes in dream quality. Recently, Rozen and Soffer-Dudek (2018) found that occurrence of teeth dreams (one of the most common dream themes reported by 39% of the population; Yu, 2010), correlated with the experience of dental irritation, but not with other measures of sleep disturbances or psychological distress.

In sum, we claim that cortical processing of bodily sensation continues during sleep and influences dream generation. Of course, sensation alone does not explain dreaming. A fascinating video of an RBD patient ‘smoking’ his pulse oximeter shows how sensation is interpreted by the dreaming mind: from the perception of pressure around the finger, this particular individual with a history of smoking dreams of enjoying a cigarette (video can be found in Oudiette et al., 2009). The dream emerges in a co-creative manner: real body sensation contributes to dream generation just as individual experience shapes dream narrative, which manifests in further body sensation, and so on, completing the dreaming circuitry. We further explore the role of individual experience in shaping dream imagery in the next section.

#### 1.2.2. Top-down sources of dreaming

Neurocognitive accounts of dreaming agree that memory shapes the content of dream experience in a top-down manner. Dreams are affected by everything from basic perceptual and semantic knowledge, to emotional and autobiographical memory, and socio-cultural and evolutionary biases. Patterns of memory activation studied in waking cognition likewise influence the process of dream generation, including the tendency to better recall recent memories (recency effect) and associative priming of conceptually related memories. Nielsen (2017) examined such patterns in microdreams, also called hypnagogic images-brief dream snippets occurring at sleep onset. Despite lasting only seconds, microdreams reveal a process of sense making that may permeate all dream generation. In microdream examples, the mind seems to instantaneously integrate real perceptions with recent experiences and associated memories into a cohesive image.

“[Image:] A heavy door made of wood suddenly swings open to the R and slams against the corner of a counter top. [Reality:] The conference speaker made a thudding sound by hitting the microphone…A slide on the screen…depicted a closed, large, brown wooden door.” (example of a microdream, Nielsen, 2017)

In this example, the image of a door slamming captures the real sound of a microphone thudding. The image is primed by the recent memory of a door depicted on a slide, and related semantic and episodic knowledge that doors make a sudden noise when slammed. Similar sense making is evident in studies of lucid dreams, where audio or visual stimuli become contextualized in the dream environment (LaBerge & Levitan, 1995). For instance, when presented with flashing LED lights one lucid dreamer reports, *“I could tell when the red light came on because it got hot and the sun got brighter”* (Carr, Konkoly, Mallett, Edwards, Appel, & Blagrove, 2020). In another example, an audio cue was contextualized in the following dream: *“I was shopping in a supermarket… I could hear the beeping and it was like I was getting loads of messages on my phone telling me what to buy … things like, ‘buy some biscuits’. ”* To make sense of audio or visual stimulation, the dream relies on knowledge of how such perceptions have arisen in similar environments in the waking world.

Thus, a major contributor to sense making lies in interpreting current experience by drawing on similar experiences from the past. Researchers have even explored the temporal patterns of memory incorporation into dreaming. For instance, microdreams often incorporate at least one recent memory trace, but then link it with more distant past experiences and general semantic knowledge (Stenstrom, Fox, Solomonova, & Nielsen, 2012). Solomonova, Stenstrom, Paquette, and Nielsen (2015) assessed the experience of spending a night in the sleep laboratory as a memory likely to be processed in dreams. One participant reported in the morning: *“I wake up and get out of the laboratory bedroom. [It] is exactly the same as I saw it yesterday…somebody is taking the electrodes off my head.”* The frequent incorporation of memory from the prior day was coined the “day residue” by Freud (1965). But memories also show delayed incorporation into dreaming, particularly after 7–9 days (the “dream-lag” effect; Nielsen & Powell, 1992). For instance, in Solomonova et al. (2015) one participant reported the following, seven days after sleeping in the laboratory: *“I am being admitted to a hospital because I am unable to remember my dreams…A team of doctors stands over the gurney telling me that I must be hospitalized.”* Hartmann (1996) proposed that recent experiences become connected over time with more distant associated memories through dreaming, and suggests emotion is a major driving force behind these associative connections. In this way, dream images connect and embody the mutual feelings of experiences. Along these lines, evidence shows that dreamers rely on “feelings of knowing” in order to recognize dream images and characters despite varied and changing appearances (Skrzypińska & Slodka, 2014), suggesting the feeling provides meaning to associatively constructed images, composites of faces and places from memory.

Another example of the sense making process can be observed in the case of sleep paralysis. Episodes of sleep paralysis involve temporary inability to speak or move while falling asleep or upon waking. These episodes are often associated with a sense of pressure on the chest and the “felt presence” of an entity in the room, typically threatening in nature (Solomonova, Frantova, & Nielsen, 2011). The sense of paralysis and pressure on the chest can be neatly explained by the muscular atonia and shallow breathing pattern characteristic of REM sleep (Cheyne, Newby-Clark, & Rueffer, 1999; Cheyne, Rueffer, & Newby-Clark, 1999). Yet, the felt presence takes forms that make sense according to cultural norms: demons, devils, witches, ghosts or aliens (Olunu et al., 2018; Solomonova, 2018). Commonly cited is “the Old Hag”, a figure from Newfoundland folklore of a wretched travelling spirit who sits on the chest of the sleeper (Hufford, 1982). In the United States, felt presence provides one explanation for alien abduction accounts (McNally & Clancy, 2005) and in Egypt, the “Jinn”, a supernatural demon from Islamic mythology appears (Jalal, Simons-Rudolph, Jalal, & Hinton, 2014), whereas Chinese adolescents interpret their experience as ghost oppression (Ma, Wu, & Pi, 2014).

While sleep paralysis is a parasomnia, and therefore may not be representative of dreaming, it demonstrates how cultural biases can shape individual imagery. Other empirical work supports that dream content can be influenced by individual current concerns, a history of adversity or trauma, or childhood attachment style, among others (for review see Domhoff, 2017), although a review is beyond the scope of this paper. In general, dreaming draws on numerous past experiences to create psychologically meaningful imagery, including recent to remote experiences, emotional to episodic memories, and cultural to evolutionary biases. And the complete dreaming circuitry allows a full being-in-the-world, in which each idiosyncratic body—musculature, cardiovascular and sensory systems—interacts with each individual world—social, emotional, conceptual, and more. Given the idiosyncratic nature of dream generation, we focus on manipulating simple attributes of sleep and dreaming rather than attempting to control entire dream narratives (see Section 2.1). Therefore, we now review research on sleep neurophysiology and associated dream content to reveal functional targets of dream engineering.

### 1.3. Functional stimulation of sleep and dreams

#### 1.3.1. Functional targets of dream engineering

Dream content consistently differs between the various sleep stages, as numerous laboratory studies have shown using polysomnography (PSG), in which sleep stages are classified according to patterns of electrical activity in the brain (Berry, Brooks, Gamaldo, Harding, Marcus, & Vaughn, 2012). Dreaming occurs initially in the form of brief hypnagogic imagery at sleep onset, in non-REM stage 1 sleep (NREM1 or N1). This transitional phase is characterized by a loss of EEG alpha activity and prominent theta activity (3–7 Hz; Berry et al., 2012). Recent experiences and even experimental tasks are frequently incorporated into N1 imagery (e.g., Tetris, Stickgold, Malia, Maguire, Roddenberry, & O’Connor, 2000; alpine racer game, Wamsley, Tucker, Payne, Benavides, & Stickgold, 2010) and incorporation of a learning task into N1 imagery has been correlated with improved performance (Wamsley, Perry, Djonlagic, Reaven, & Stickgold, 2010). N1 images are also described as surreal and have been associated with creativity and insight (McKellar, 1995).

NREM stage 2 (N2), which follows N1 sleep, is characterized by K-complexes (high-voltage slow waves) which are frequently coupled with sleep spindles (bursts of 12–15 Hz oscillations; Berry et al., 2012). Dream reports collected from N2 are longer than those from N1, and often contain reference to thought Speth and Speth (2018). Functionally, N2 sleep and particularly sleep spindles have been linked to declarative memory consolidation (Ruch et al., 2012; Tucker et al., 2006), and sleep spindle density correlates with dream recall (Nielsen et al., 2017). NREM stage 3 (N3), also known as slow wave sleep, is characterized by prominent delta waves (0.5–4 Hz; Berry et al., 2012). N3 is often termed deep sleep and is thought to be most important for homeostatic processes (Tononi & Cirelli, 2003, 2006); dream recall frequency is lowest from N3, with dream reports that are short and phenomenally minimal (Cavallero, Cicogna, Natale, Occhionero, & Zito, 1992). Recent findings suggest that delta activity over parietal areas is a predictor of dreamless sleep (Siclari et al., 2017); whereas fast-wave activity in the same areas, most typical of REM sleep, is a predictor of dream recall.

Finally, REM sleep is characterized by higher frequency activity similar to N1 or wakefulness, with particularly increased theta oscillations (Berry et al., 2012). REM sleep dreaming provides a rich “virtual laboratory for the rehearsal of embodied cognition” (Speth & Speth, 2018). Procedural memory benefits from REM sleep (Smith, 2001), with some evidence that motor practice during lucid dreaming corresponds with improved performance after sleep (Schädlich, Erlacher, & Schredl, 2017). REM sleep is also functionally involved in emotion regulation (Gujar, McDonald, Nishida, & Walker, 2010; Van Der Helm et al., 2011) although nightmares, intense negative dreams, can interfere with this function and cause distress in waking life (Kramer, 1991; Levin & Nielsen, 2009; Nielsen & Carr, 2017). Positive dreams may have the inverse effect, associated with improved sleep quality and subsequent mood (Carr & Nielsen, 2017). Finally, REM sleep is linked to increased insight and creativity (Cai, Mednick, Harrison, Kanady, & Mednick, 2009; Carr & Nielsen, 2015; Stickgold, Scott, Rittenhouse, & Hobson, 1999; Walker, Liston, Hobson, & Stickgold, 2002), with imagery described as hyperassociative and metaphorical, likened to a form of creative expression (Hartmann & Kunzendorf, 2013).

Overall, dream engineering techniques are informed by sleep stage neurophysiology and corresponding dream quality. Functional targets of dream engineering include improving sleep quality, enhancing memory consolidation, ameliorating emotion regulation, inspiring creativity, and augmenting motor learning. To date, several techniques have been developed in sleep laboratories to reach these aims, which we overview below.

#### 1.3.2. Stimulation techniques for dream engineering

Sensory manipulation has been used for millennia to influence dream content. The earliest reference is inscribed on the Chester Beatty papyri, found in Upper Egypt and authored c.1350 BCE. It describes a method of drawing on the hand and covering the hand and neck in black cloth prior to sleep in order to invoke the wisdom of Besa, a dwarf deity (Nielsen, 2012). Fasting has been used to trigger vivid dreams in many cultures from Egyptians to indigenous peoples of North America, and modern cultures believe spicy or dairy foods trigger vivid dreams (Nielsen & Powell, 2015). Experimentally, Dement and Wolpert (1958) collected 15 REM dream reports from individuals deprived of fluids for 24 h prior to sleeping in the laboratory, and five dreams contained thirst-related content. Pre-sleep priming with a visual stimulus is likewise effective; Goodenough et al. (1975) show that watching a stressful film prior to sleep increased dream negativity, whereas use of visual inverting prisms led to more active and vivid dreams (Corsi-Cabrera et al., 1986). Pre-sleep rehearsal of current concerns, as opposed to exposure to stimuli, also has some efficacy in incubating dreams. Barrett (1993) asked college students to think about a personally relevant problem for 15 min before sleep, and in response, 49% of reported dreams were rated as relevant to the problem, with 34% of them containing a solution. Saredi, Baylor, Meier, and Strauch (1997) similarly found that thinking of a question related to a current problem prior to sleep increased the likelihood that dream content reflected the problem. Finally, in Imagery Rehearsal Therapy, visualizing a positive ending to a nightmare prior to sleep leads to resolution of nightmares within sleep (Krakow & Zadra, 2010).

In the absence of pre-sleep manipulation, stimulation applied only during sleep can direct dream content in various ways. A spray of water on the skin (Dement & Wolpert, 1958), application of a pressure cuff to a specific limb (Nielsen, 1993), or application of electrical pulses to cause muscle contractions in a sleeper (Koulack, 1969) have each been shown to affect dream features, increasing vividness or movement sensations in dreams. In Nielsen (1993), pressure application to the leg resulted in visual-kinesthetic synesthesia, direct incorporation of pressure and squeezing sensations, and increased bodily bizarreness in dreams. Schredl et al. (2009) show that pleasant scent increases dream positivity whereas unpleasant scent increases negativity. Stimulation can also influence neural oscillations during sleep (Hennevin, Huetz, & Edeline, 2007; Stickgold, 2001). As research reveals the functional roles of specific sleep oscillations, e.g., sleep spindles being critical for memory consolidation, these rhythms become targets for entrainment. Playing bursts of oscillating white noise during N2 and N3 at 12 or 15 Hz can increase the number of sleep spindles (Antony & Paller, 2017). And applying oscillating electrical potentials to the scalp at 0.75 Hz has been used to augment slow oscillations in N3, resulting in improved memory performance the following day (Marshall, Helgadóttir, Mölle, & Born, 2006).

Finally, experimental protocols have combined pre-sleep and within-sleep stimulation to induce ‘targeted reactivation’ of specific content. In Targeted Memory Reactivation (TMR), a sensory stimulus is associated with a learning task prior to sleep, and is then re-presented during sleep to trigger associated memory reactivation (Lewis & Bendor, 2019; Rudoy, Voss, Westerberg, & Paller, 2009). For instance, presenting subjects with an olfactory cue during NREM sleep that was previously presented during an object location task enhances memory consolidation (Rasch, Büchel, Gais, & Born, 2007; Rihm, Diekelmann, Born, & Rasch, 2014). Auditory cues, including speech, can target reactivation of declarative memories and have frequently been used to enhance language learning (Oudiette & Paller, 2013). Re-presenting an auditory or odor cue during sleep can even enhance fear extinction in both mice and humans (Hauner, Howard, Zelano, & Gottfried, 2013; Oudiette, Antony, & Paller, 2014).

Targeted reactivation techniques can also be applied to dreaming. For instance, Targeted Dream Reactivation (TDR) pairs a stimulus with pre-sleep priming or dream incubation, and re-presents the stimulus during sleep to trigger relevant dream content. An early example combined pre-sleep thirst with presentation of liquid-related words in REM sleep: Dreams about liquids increased, and those who dreamt of quenching their thirst drank less once awake than those who dreamt of being thirsty (Bokert, 1968). De Koninck and Koulack (1975) presented a stressful film prior to sleep, then re-presented the film music during sleep: this led to increased film elements in dreams, and increased emotionality the following day. Induction of lucid dreams has also been facilitated through audio and visual stimulation during sleep (e.g. via DreamLight mask, LaBerge & Levitan, 1995). Within a Targeted Lucidity Reactivation (TLR) protocol, audio and visual stimuli are associated with a lucid mind-state prior to sleep, and then re-presented during REM sleep; this technique induced lucid dreams in 50% of participants in a morning nap (Carr et al., 2020). In addition, novel protocols are emerging all the time; for instance, Targeted Dream Incubation creatively combines components of targeted reactivation with intentional incubation at the hypnagogic border between sleep and wake (Horowitz, Cunningham, Maes, & Stickgold, 2020). Applying targeted protocols to dream content has potential for enhancing sleep functions, e.g., incubating creativity or ameliorating mood via pleasant dreams.

In sum, techniques from rhythmic entrainment to targeted reactivation, pre-sleep priming, dream incubation and dream direction each offer a sense that stimulation of specific sleep and dream content is both experimentally feasible and potentially functional (see Table 1). We posit that 1) an understanding of dreaming in circuit with the body and 2) an application of contemporary technology from the field of HCI can make the systematic induction of themes into simulated dream worlds a reality. HCI researchers have spent decades exploring how to generate simulations through a variety of sensory stimulation, which have potential to be adapted to dream engineering purposes.

## 2. Simulating worlds through sensory stimulation

The field of HCI has a storied history of building devices to create and alter experiences of simulated worlds. Virtual Reality (VR), specifically, is a medium that allows the user to experience a simulated but immersive reality, typically created through Head-Mounted Displays or projected environments. A successful VR experience relies on maintaining the illusion of being in the simulated environment, which is accomplished by continuous injection of sensory information via interactive devices, especially wearables. Each of these devices is designed for a particular sensory manipulation (e.g., one particular wearable might simulate the smell of an experience); therefore, we posit that these interactive devices are *also* reliable candidates to engineer the sensory experience of the dreamer’s simulated world.

The perspective of HCI researchers on creation and maintenance of illusory worlds can likewise enrich neuroscientific accounts of dream generation. Existing theoretical dream science portrays dreaming as a form of simulated reality (Hobson et al., 2014; Nielsen, 2010; Valli & Revonsuo, 2009); Hobson et al. (2014) called dreams “an innate virtual reality”. The perspective of dream theorists has been primarily to deconstruct dream features and memory sources and identify similarities with waking reality. HCI researchers instead approach the building of illusory worlds from the ground up, using technology to construct plausible and immersive realities. The mechanisms that enable this illusion generation could thus shed light on the organic mechanisms that make the dream world immersive and plausible.

Many VR researchers have studied the underlying brain mechanisms that enable illusions in VR. Gonzalez-Franco and Lanier (2017) proposed a model explaining the cognitive and perceptual mechanisms of simulation generation: (1) Bottom-up multisensory processing (Blanke, 2012; Calvert, Spence, & Stein, 2004); (2) Top-down prediction manipulations (Haggard, Clark, & Kalogeras, 2002); and, (3) Sensorimotor self-awareness frameworks (Gallagher, 2000). They also propose three main types of illusions which account for simulation maintenance, based on previous VR work (Spanlang et al., 2014): (1) Plausibility illusion – participants feel that the events happening in the virtual world are real; (2) Embodiment illusion – participants feel that they inhabit a virtual person; and, (3) Place illusion – participants feel they inhabit a virtual location. These mechanisms of generating and maintaining illusory worlds in VR echo the accounts of dream generation described above, lending theoretical support for the translation of technologies from HCI to dream engineering.

### 2.1. The sensory interface in dreaming and VR

In both dreaming and VR simulations there is limited access to the “real world”. In the field of HCI, achieving more realistic simulations (as well as more immersive virtual environments) required researchers to tackle this limitation. The typical approach is to develop stimulation technologies that allow users to perceive more sensory cues while interacting in a simulated world; these cues add up to immersion, a sense of belief in the VR experience, which is caused by a predictability of the effects of the virtual world in the user’s senses. While the early decades of VR research focused on attaining visual realism, today’s research focuses instead on the other senses, with a particular emphasis on somatosensation (touch, forces), vestibular sense, thermosensation and olfaction. These are particularly interesting for dream research because of the aforementioned close analogy between virtual and dream worlds.

Nevertheless there are obvious constraints to simulating dream worlds. First, unlike in VR, sensory processing in sleep is limited by both gating and arousal mechanisms. Gating mechanisms act to selectively filter sensory information during sleep and vary by sleep stages and type of stimulation (Andrillon & Kouider, 2019). For instance, cortical processing is selectively amplified for relevant as opposed to irrelevant speech (Legendre, Andrillon, Koroma, & Kouider, 2019) and speech related to current concerns, but not unrelated speech, has been shown to influence dream content (Hoelscher, Klinger, & Barta, 1981). At the same time, sleep is a fragile state, and an abundance of sensory stimulation will lead to microarousals or full awakenings. A second limitation that distinguishes dreaming from virtual simulations is the level of control the experimenter has over the simulation. In VR, an entire multisensory world can be scripted into a predefined narrative. In dreaming, the multiplicity of idiosyncratic autobiographical memories, recent experiences, socio-cultural and evolutionary pressures cause the dream to emerge in a relatively unpredictable manner.

Thus, continued basic experimental research is necessary to further unveil which stimuli cross the threshold of sleep, whether these stimuli enter into dream content, and how the impact of stimuli on dream content varies within and between individuals. The same auditory or olfactory stimuli could transform into various dream images based on the user’s memories, cultural background, emotional state, etc. These limitations at a physiological and phenomenological level guide our approach to dream engineering: we aim to affect simple attributes of dreaming via sensory stimulation, e.g. to increase movement or ameliorate emotion without attempting to control the manifest content of such dreamed movement or emotion. We here discuss simple sensory HCI technologies that, we believe, can be used for dream engineering.

### 2.2. Stimulating the sense of touch and force (Somatosensation)

HCI researchers have built a series of interactive devices that stimulate the haptic sense in order to convey the physicality of the virtual world, which could be applied to incorporate tactile stimulation in dreams (Brooks, 1999). There has been progress on two fronts: (1) simulating the cutaneous qualities of interacting with lightweight objects, such as contact with surfaces or textures (Culbertson, López Delgado, & Kuchenbecker, 2014), and (2) simulating the proprioceptive side of interaction with objects that arises from their weight, i.e., the forces that arise when we push or lift a heavy object (Gu, Zhang, Sun, Bian, Zhou, & Kristensson, 2016).

Vibration is a common modality in VR haptics because vibration actuators are cheap and relatively small, fitting into wearables such as gloves (Burdea, 2000) or vests (Lindeman, Yanagida, Noma, & Hosaka, 2006). Generally, vibration emulates touching an object (Kron & Schmidt, 2003) or conveys the texture of objects (Culbertson et al., 2014), with high frequency vibration conveying smooth texture, and low frequency conveying rough textures. Vibration is not typically used to simulate the sense of pressure, although devices can create a pseudo sense of directional pressure using an asymmetric vibration pattern (Rekimoto, 2013). Other tactile devices create small forces sufficient to displace the user’s skin laterally (Chen, Anderson, Walker, & Besier, 2016), e.g. *Pneumatic Gloves* contain air pockets that inflate when the user’s fingertips touch a virtual object (Benali-Khoudja, Hafez, Alexandre, & Kheddar, 2004).

While technologies for simulating contact with objects has been maturing over the past decades of HCI research, simulating large forces that arise from interactions with objects is still a focus of much VR research. The main approach employs mechanical actuation, such as pulling on tethers attached to the user (*SPIDAR*; Murayama et al., 2004), e.g. to simulate hitting a virtual baseball (Jeong, Hashimoto, & Makoto, 2004). While mechanically actuated devices offer high precision, they are still large and cumbersome devices. Therefore, HCI researchers explored actuating the user’s muscles with electrical impulses as a means of creating force feedback. This technique, called electrical muscle stimulation (EMS), originated in the field of rehabilitation medicine where it is applied to regain lost motor function (Strojnik, Kralj, & Ursic, 1979). Tamaki, Miyaki, and Rekimoto (2011) used EMS in an interface that guided users in learning a new instrument and Pfeiffer, Dünte, Schneegass, Alt, and Rohs (2015) used an EMS-based device to steer users while walking. Unarguably, EMS’ largest application area in HCI is as a wearable to create force-feedback in virtual simulations: to add forces to mobile devices (Lopes & Baudisch, 2013); to render the sensation of a ball hitting a racket in an augmented reality tennis game (Farbiz, Yu, Manders, & Ahmad, 2007); to simulate touching virtual walls and objects (Lopes, You, Cheng, Marwecki, & Baudisch, 2017; Lopes, You, Ion, & Baudisch, 2018); and to simulate the sensation of hitting or being hit in VR sports, such as boxing (Lopes, Ion, & Baudisch, 2015). The latter wearable device uses a combination of tactile stimuli (a solenoid tapping the skin) and force feedback (electrical muscle stimulation) to create more realistic illusions of feeling a virtual object, i.e., not only touching the object’s surface but also feeling the object’s inertia.

#### 2.2.1. Somatosensory stimulation in dream engineering

While the effect of somatosensation on dream content has been described earlier, the use of small wearables presents an avenue for conducting novel studies potentially in home users. For instance, *Pneumatic Gloves* provide a wearable similar to the pressure cuff used in (Nielsen, 1993).

Importantly, the application of EMS presents a relatively new avenue of dream direction (though see Koulack, 1969). EMS application could functionally simulate twitches that naturally occur during REM sleep. Recent work suggests that sensorimotor feedback from twitches during REM sleep is critical for development and maintenance of motor coordination (Blumberg, Marques, & Iida, 2013). An open question is whether targeted application of low-level EMS could potentially enhance motor learning during sleep, in parallel to the rehabilitative effects of EMS on motor function in wake (Strojnik et al., 1979). Further, EMS could trigger motor imagery during sleep, which likewise has been associated with enhanced motor learning (e.g., in lucid dreams, Schädlich et al., 2017). In one recent study, Debarnot, Perrault, Sterpenich, Legendre, Huber, Guillot, and Schwartz (2019) found that waking motor imagery practice during arm immobilization led to increased subsequent REM sleep and improved adaptation the next day. The use of EMS to augment motor dream imagery thus offers an intriguing application of dream engineering for physical rehabilitation.

As an initial pilot test of the concept, we applied EMS to one pilot subject (approved by MIT’s Committee on the Use of Humans as Experimental Subjects) using an FDA approved electrical muscle stimulator device with a maximum voltage and current of 70 V and 0.72 mA respectively. EMS was applied during a nap on the upper and lower calf muscle after at least 5 min of REM sleep, defined as minutes in which eye movements exceeded 10 eye movements per minute (Takahashi & Atsumi, 1997). The subject was awakened for a report after the first stimulation, then the procedure was repeated for a second trial. The EMS application did not waken the subject, and dream reports obtained after awakening did contain reference to the limb stimulation. The first report contained reference to running: *“Was like a beach…just looking at them, the rocks…I can see my feet…I had a small image of running in a field. And then feeling the grass hit on my feet.”* In the second report, the stimulation seemed to transform into an audiovisual experience in the dream: *“I didn’t get any dreams until I started feeling the device. Yeah it was cool, at some point you can anticipate the increase of the, you know, da-da-da-da-da-da [‘the shock’] and then once it started to get stronger you kind of will be waiting for like boom this is the peak and then at the peak you get an image.”* Although preliminary, we suggest EMS as a technology for REM sleep dream direction is worthy of future investigation.

### 2.3. Stimulating the sense of temperature (thermoception)

To improve the realism of VR experiences, researchers in the field of HCI have been developing and adding more haptic sensations. More recently, attention has been given to simulating the temperature of a virtual experience (Peiris, Peng, Chen, Chan, & Minamizawa, 2017). This is typically achieved by means of heating or cooling the air around the user’s skin, e.g., using air conditioning units (Xu, Kuroda, Yoshimoto, & Oshiro, 2018) or heat lamps (Hülsmann, Fröhlich, Mattar, & Wachsmuth, 2014) or by directly heating/cooling the skin using thermoelectric elements, also known as Peltier elements (Peiris et al., 2017). The latter are especially relevant since they are smaller and fit into wearables, i.e., they attach directly to the user and not to the user’s environment. Peltier-based thermal devices create a temperature difference across two plates in the presence of an electric current. These thermal devices have been used for realism and for transmitting information in general purpose interfaces, for instance adding thermal navigation cues on a steering wheel (Vito, 2019). The *ThermalBracelet* (Peiris, Feng, Chan, & Minamizawa, 2019a, 2019b) attached Peltiers to the user’s wrist to explore eyes-free feedback, and Peiris et al. (2017) added Peltiers to a VR headset, creating temperature sensations directly on to the user’s face. *Season Traveller* (Ranasinghe & Tung, 2018) utilized heating elements on the back of the user’s neck combined with an olfactory display to generate realistic scented environments; lastly, *Thermotaxis* (Narumi, Tomohiro, Seong, & Hirose, 2009) used Peltier elements attached to the user’s ears to render temperature changes.

Alternative technologies that stimulate thermoception include: hydraulics (i.e., pushing hot/cold liquids through tubes that are in contact with the user’s skin; Han, Anderson, Irani, & Grossman, 2018), gel packs (Jain et al., 2016), resistive heating (Wettach, Behrens, Danielsson, & Ness, 2007) and, more recently, a thermal illusion based on projecting scents, such as mint or capsaicin, that are perceived as cooling or warming by the user’s trigeminal nerve when inhaled (Brooks, Nagels, & Lopes, 2020).

#### 2.3.1. Temperature in dream engineering

Sleep is closely tied to the circadian rhythm of core body temperature (Lack & Lushington, 1996). To date, researchers have explored passive body heating through the skin to decrease sleep onset latency (Raymann, Swaab, & Van Someren, 2007) and warming of the periocular skin to improve subjective sleep quality (Sakamoto et al., 2017) and even increase delta power during sleep (Igaki et al., 2018). The authors of the latter study developed a heat- and steam-generating sheet to warm the skin, either around the eyes, neck or abdomen, which proved effective for improving sleep quality.

While warmth helps individuals to fall asleep, core body temperature drops during deep sleep, with optimal sleep temperature reported to be somewhere between 60 and 68 degrees Fahrenheit (16–20 degrees Celsius) (Harding, Franks, & Wisden, 2019). Thus, cooling the body has also been explored as a means of enhancing deep sleep. For instance, the *Kryo Chilipad* is a temperature-controlled cooling mattress topper that can cool to 60 degrees Fahrenheit using a water-based thermoregulator (Inc, 2019).

HCI concepts, such as the development of small actuators that can heat or cool in a matter of milliseconds (Peiris et al., 2017; Peiris et al., 2019a, 2019b), or even stimuli such as scents that are associated with sensations of heating or cooling (such as capsaicin or peppermint), provide simple technologies for dream engineering. Ideally, temperature devices with application to sleep would be able to heat or cool depending on need and would be safe and easy to use throughout the night. One possibility is integrating wearable HCI devices with sleep tracking software. In fact, warming/cooling scents could be presented via *Essence*, an olfactory device described later.

### 2.4. Stimulating the sense of balance (vestibular)

**Fig. 1.**
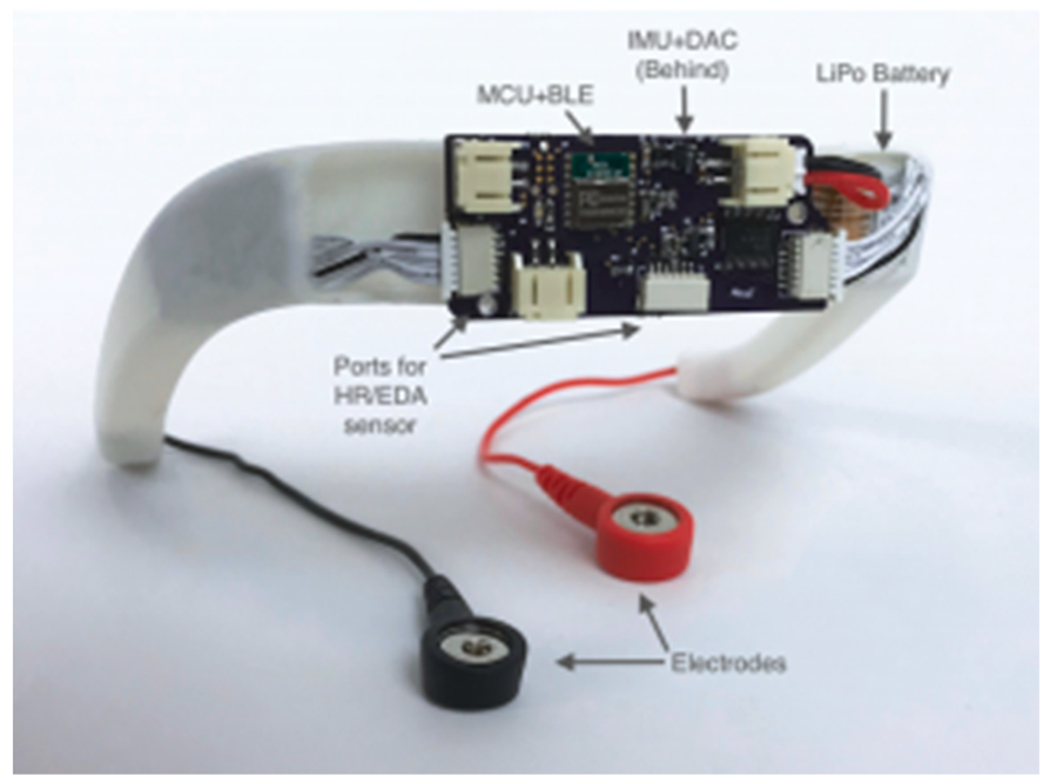
Electrodes attached behind the ear are used to transmit a small electrical current to the vestibular system, providing proprioceptive information to the user.

Vestibular proprioception consists of both the sense of movement and the position of the body, creating a sense of bodily balance called the vestibular sense. A good amount of progress has been made towards simulating the body’s vestibular sense in VR, especially walking. Our approach (Sra, Jain, & Maes, 2019) to simulating movement in virtual environments is to provide proprioceptive feedback related to virtual motion by directly stimulating the user’s vestibular system (see Fig. 1; based on Fitzpatrick, Burke, & Gandevia, 1994). The Galvanic Vestibular Stimulation system sits on a user’s neck with electrodes attached behind the ear, on the mastoid bone of the user. A small amount of electrical signal is delivered in a controlled manner, giving balance signals across the vestibular system, which corresponds to virtual movements. These balance signals significantly increase the sense of realism in the VR experience, and the system enables induction of a full body sensation of motion in 4 relative directions (forward, backward, left and right).

#### 2.4.1. Vestibular sense in dream engineering

Electrical stimulation of the vestibular sense opens up opportunities for rocking subjects to sleep with wearable electronics and influencing sense of balance in simulated dream bodies. Sleep neuroscience research has shown that sensations of swinging can modulate physiological parameters of sleep, easing sleep onset and inducing a sustained boosting of slow oscillations and spindle activity (Bayer et al., 2011). It is proposed that the swinging motion exerts a synchronizing action in the brain that reinforces endogenous sleep rhythms—yet this study required physically rocking a whole bed. Our Galvanic Vestibular Stimulation device could potentially deliver rocking sensations without the construction of a mobile bed, enabling targeted direction and timing of vestibular sensations in a small wearable device. Other devices have shown simple movement signals can significantly influence sleep. The *Breathing Bear* (Ingersoll & Thoman, 1994) uses rhythmic movement stimulation from an actuated toy to entrain infant breath, easing sleep onset. Haptic interfaces such as *NightShift* have been used to alleviate sleep apnea by delivering vibration to make users change from unhealthy body positions during sleep (Scarlata, Bartoli, Santangelo, Giannunzio, & Incalzi, 2015). Thus, bodily movements influence both sleep physiology and quality, and are a viable target for sleep manipulation technologies.

### 2.5. Stimulating the olfactory sense

The history of olfactory technological research can be dated to the late 50’s when scents were released during the viewing of films to associate certain smells with scenes of a movie (*Smell-O-Vision* and *Sensorama;* Heilig, 1961). A decade ago, the HCI research community started looking into the challenges and possibilities for smell-based technology. Recent HCI research and product developer efforts have focused on enabling scent to become part of digital communications. Most systems use off the shelf aromas in their prototypes, focusing research efforts on the device itself rather than facing the chemical engineering challenge of capturing odors. Although limited work has been published in the field of olfactory displays for VR, some researchers (Ischer et al., 2014), showed that the addition of smell in VR significantly enhances the sense of immersion. Li and Bailenson (2018) demonstrated that olfactory and haptic cues in VR have satiation effects. Pleasant ambient odors have also been used to relieve stress and improve relaxation when coupled with VR (Carskadon & Herz, 2004), or as a biofeedback tool for mindfulness and meditation (Amores, Dotan, & Maes, 2019, Amores, Wang, Dotan, & Maes, 2019). Keller, Kouzes, Kangas, and Hashem (1995) created an electronic nose that can identify certain odors and transmit olfactory information for telepresence VR systems. Tijou, Richard, and Richard (2006) explored the use of olfactory cues for educational purposes using large-scale fan-based devices while students learned the structures of organic molecules. More recently, Radvansky and Dombeck (2018) developed the first olfactory VR system for mice, to study odor-guided virtual navigation behavior. Such research will open new translational opportunities to study olfactory learning and sleep in humans.

#### 2.5.1. Scent in dream engineering

In comparison to sight or audio, odors presented during sleep are less likely to cause arousal. Olfactory information passes directly from the olfactory epithelium in the nose to the olfactory bulb in the forebrain and then on to the olfactory cortex, whereas other sensory modalities go through the thalamus which is linked to arousal. The awareness of odors during sleep is relatively low, although the use of very putrid, fish-like odors like Pyridine, as well as arousing scents like peppermint can wake people up given increased intensity (Stuck et al., 2007). These two types of smells activate the trigeminal nerve, and therefore, even if the person is entirely anosmic (incapable of smelling), they can differentiate these smells based on physical sensation. In contrast, using non-trigeminal olfactory stimuli, an odor with increased intensity does not cause nocturnal arousals (Stuck et al., 2007). Although odors might not lead to awakenings, they are still processed by the brain (Badia, Wesensten, Lammers, Culpepper, & Harsh, 1990). Some studies suggest that smells can modulate the circadian rhythm (Granados-Fuentes, Tseng, & Herzog, 2006) or reduce sleep onset latency and improve sleep quality (Field et al., 2008). A study by Arzi et al. (2010) showed how odors influenced respiration during sleep: decreased inhalation and increased exhalation following an odor release. Another interesting study found that triggering scents during sleep increased delta frequencies and sleep spindles proportional to smell duration (Perl et al., 2016).

Other researchers have assessed the impact of smell on dreams. Schredl et al. (2009) compared the results of using the smell of roses, rotten eggs, or placebo on dreams reported from REM sleep. Dream emotions were more positive in the case of the rose scent, and negative with that of rotten eggs. The same authors conducted a more recent study showing that if an odor is associated to images during wakefulness, and re-presented while in REM sleep, subjects report having dreams of those images (Schredl, Hoffmann, Sommer, & Stuck, 2014). Rasch et al. (2007) and Klinzing et al. (2018) used odor cues to enhance learning of visuospatial locations via targeted memory reactivation during N2/N3, which enhanced memory retrieval of 2D object-locations after sleep. In these studies, researchers used a full PSG setup and a nasal mask or nasal cannula connected via long Teflon tubes to a computer-controlled olfactometer. The scent-release device is placed in an adjacent room because the air pumps used to release scent are noisy. Thus, current olfactometers are expensive and not wearable; therefore, HCI researchers (Amores, 2016) created a miniaturized, silent, wearable olfactometer, the *Essence* device, which we present in detail in the following as we believe it is a particularly useful device for dream engineering.

#### 2.5.2. The Essence device

**Fig. 2.**
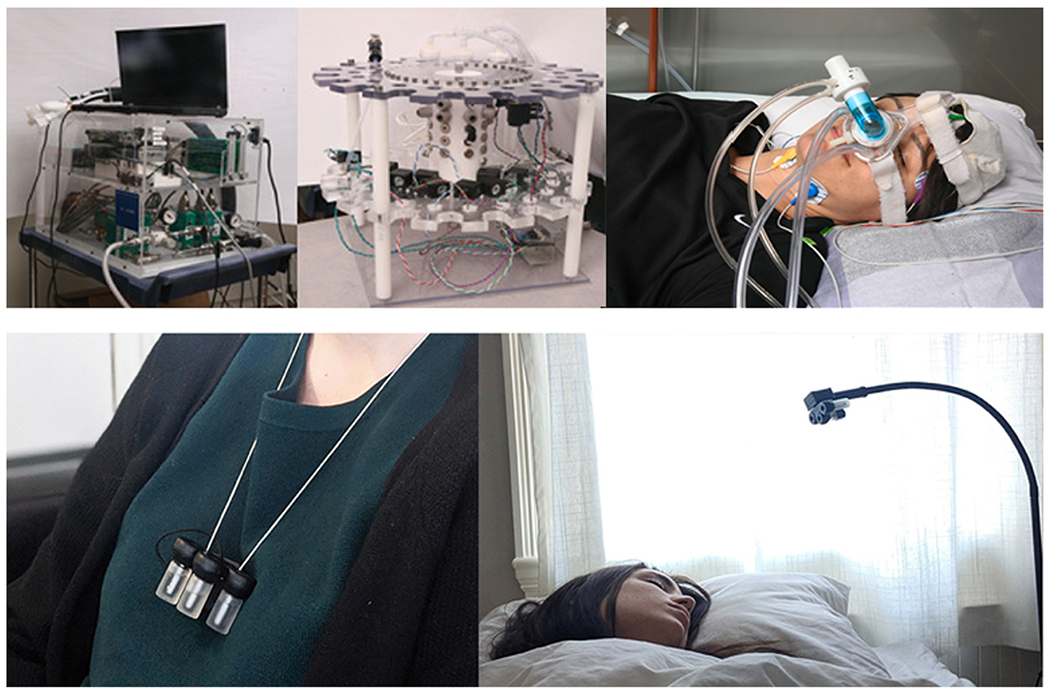
(top) Traditional PSG and olfactometers used for sleep studies. (bottom) The Essence prototype can be worn during the day and clipped to a flexible holder at night. The scent parameters and position can be adjusted to the user’s or experimenter’s preferences.

*Essence* is a wearable that can release bursts of scent based on physiological information of the user. It can be worn as a necklace or a clip and can release odors based on heart rate and brain activity (Fig. 2, Amores & Maes, 2017). The device is wireless and connects to physiological sensors as well as the *Muse* EEG headband; it monitors heart and breathing rate from sensors integrated into the device (Amores, Hernandez, Dementyev, Wang, & Maes, 2018). The device can release multiple fragrances that can be remotely controlled by an Android app, and can release scent based on individual parameters, for example ‘if HR greater than 80, release pleasant scent’. The system can connect to the *Muse* EEG headband and can incorporate sleep-staging algorithms described in this paper (Koushik, Amores, & Maes, 2019). The device has been successfully tested with more than 100 participants for use during the day or in combination with VR for relaxation (Amores, Richer, Zhao, Maes, & Eskofier, 2018).

Essence was proposed as a “sleep user interface” with olfactory cues to open a new interaction opportunity in HCI (Amores, Dotan, & Maes, 2019, Amores, Wang, Dotan, & Maes, 2019). The device could be used to release pleasant scent based on physiological indicators of nightmares; or used as a device for targeted reactivation protocols. A pilot experiment was conducted with a participant who suffered PTSD, with the intention of positively mitigating traumatic nightmares using olfactive and sound cues during slow-wave sleep (Amores, 2016). For this pilot case study, unpleasant trauma-related sounds were paired with pleasant odors or presented without odor; following sleep all sounds were rated as less arousing and more pleasant than prior to sleep, though this difference was larger for sounds that had been paired with pleasant odors. While preliminary, our ultimate goal is to open new opportunities for sleep and HCI researchers to conduct at-home sleep-olfactory studies.

### 2.6. Stimulating the auditory sense

Audio has always been used in tandem with visual stimulation in VR simulations. Spatialized audio has been used to increase sense of immersion in simulated virtual space (Naef, Staadt, & Gross, 2002), while real-time audio has been used to enable sense of presence for virtual collaborations (Monahan, McArdle, & Bertolotto, 2008). Technologies used for audio have ranged from headsets to bone-conduction speakers (Walker & Lindsay, 2005). A series of technologies have also been built to probe and alter the dreaming mind using audio. Simple apps, like *SleepBot*, provide interfaces for dream logging in the morning after a night of sleep via text or audio recording (Ong & Gillespie, 2016). One device that builds on these simple audio interfaces is Dormio (Horowitz, Grover, Reynolds-Cuéllar, Breazeal, & Maes, 2018), which we present in the following as a useful device for dream engineering.

#### 2.6.1. The Dormio system

**Fig. 3.**
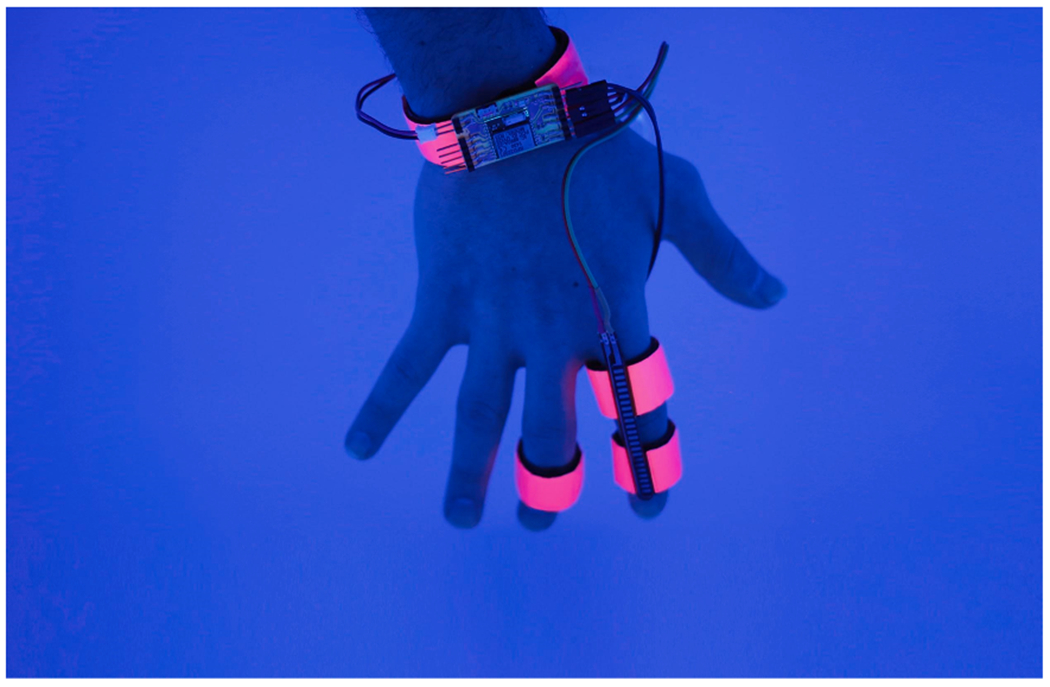
The Dormio, design led by Tomás Vega, and handworn system, collaboration with Oscar Rosello, dorsal side. Photo credit Oscar Rosello.

The Dormio system is a combined sleep tracker and dream incubator, focusing on incubation of sleep onset dreams using auditory semantic cues. The Dormio device is wrist-worn (see Fig. 3) with an associated app used to communicate with users and record dream reports via laptop or cellphone. The system tracks sleep onset and then initiates serial awakenings, inserting a dream incubation theme during each inertia-laden awakening, creating a serial dream incubation paradigm.

To achieve this, Dormio uses three physiological indicators of sleep onset in conjunction. First, users are asked to gently close their hand when they lie down to sleep, allowing a flex sensor to monitor progressive loss of muscle tone via hand opening. This is a passive behavioral measure of sleep onset (Kelly, Strecker, & Bianchi, 2012; Prerau et al., 2014), as loss of muscle tone is temporally tied to onset of hypnagogic imagery. Recent papers have also demonstrated that drops in heartrate and shifts in electrodermal activity (EDA) coincide with loss of muscle tone to confirm descent into hypnagogia (Herlan, Ottenbacher, Schneider, Riemann, & Feige, 2019; Ogilvie, 2001). The user’s heart rate is monitored on the middle finger, muscle tone is tracked using a sensor wrapped around the index finger, and EDA is measured between two electrodes placed on the bottom of the wrist. The Dormio glove is designed to be free of wires, breathable, lightweight and comfortable for sleep.

The Dormio app (ios, web) provides an interface for users to control their hypnagogic experiences. There are features for recording the incubation prompts, and for inputting the desired number of hypnagogic rounds. On each awakening, the user verbally reports their dream, which is recorded by the app, and a silence detection feature stops the recording once the report is finished. To gather and store sensor data, the Dormio Web App makes use of OpenSleep, a framework built for biosignal tracking and analysis. Results in Horowitz et al. (2018) and Horowitz (2019) show that Dormio increases direct inclusion of target words in reported dream content, and suggests that inclusion of target words in dreams is linked to improved performance on a range of creativity tasks related to these targets.

### 2.7. Physiological tracking for HCI

A key step in creating effective virtual world illusions is tracking behavior and physiology of the user, so that stimulation timing and type is appropriate to increase immersion (predictable, believable, etc.) by being coherent with virtual simulations. In addition to a VR visual Head-Mounted Display (HMD), sensors can be added to enable real-time monitoring of a user’s physiological and cognitive state, for instance, heart rate and EDA can be physiological indicators of emotional arousal (Sequeira, Hot, Silvert, & Delplanque, 2009), EEG can provide data regarding cognition (Klimesch, 1999), and facial EMG can be used to detect emotional facial expressions (Van Boxtel, 2010). HCI research has developed hardware and software solutions to provide accurate, real-time information regarding a user’s response to content in a virtual environment in devices that are affordable, wearable, and easy-to-use. For instance, even though the main method for automatic emotion recognition is audio-visual analysis (De Silva, 2004; Wang & Guan, 2008), HCI has looked to EMG to decode emotional expressions (Branco, Firth, Encarnação, & Bonato, 2005; Mahlke & Minge, 2006), removing the need for a front-facing camera, and allowing expression detection regardless of illumination or noise (essential for translation to sleep applications).

#### 2.7.1. Sleep tracking

Although PSG is the gold standard for sleep staging, it is both expensive and impractical for users at home (Ravichandran, Sien, Patel, Kientz, & Pina, 2017). In recent years, there has been an increase in development of low-cost sensors aimed at tracking sleep outside of the laboratory without the need of a PSG setup (de Zambotti et al., 2016). Research shows nine out of ten Americans report using a technological device in the hour before sleep, facilitating the introduction of technological interventions for the bedroom (Gradisar et al., 2013). Many sleep trackers are based on mobile apps, although wearables, smart pillows and smart sheets exist as well (Heise, Rosales, Sheahen, Su, & Skubic, 2013; Lawson et al., 2013; Schreiner & Staresina, 2019). For example, Tal, Shinar, Shaki, Codish, and Goldbart (2017) presented a novel contact-free, under-the-mattress piezoelectric sensor that senses heart, breath and body movement patterns and shows 90.5% sleep stage detection accuracy. Sensors like the Oura ring offer non-intrusive, wireless sleep tracking with adequate staging sensitivity compared to PSG (de Zambotti, Rosas, Colrain, & Baker, 2019). These trackers show promise for users getting information about their sleep, but without the real time sleep staging which is necessary for interventions or interaction in sleep.

The Nightcap home sleep monitoring system enabled a host of important home-based studies in the 1990s (Ajilore, Stickgold, Rittenhouse, & Hobson, 1995; Cantero, Atienza, Stickgold, & Hobson, 2002; Mamelak & Hobson, 1989; Stickgold & Hobson, 1994; Stickgold, Pace-Schott, & Hobson, 1994). The Nightcap used eyelid, eyeball and head movement to track sleep onset, REM sleep and deep NREM sleep in real time and trigger automatic collection of dream reports by sleep stage, with only a 5–10% decrease in accuracy in sleep state identification compared to PSG (Ajilore et al., 1995). However, it relied on custom and uncomfortable sensors stuck to the eyelid and remained wired with a bulky amplification circuit, neither of which is ideal for sleeping settings. We recently modernized the Nightcap into a device called *Masca* (Vega, 2019), which uses piezoresistive fabric sensors to detect eye movement; these sensors can be placed in an eye mask instead of affixed to the eyelid. Custom-built miniature circuit boards allow signal amplification and measurement of head movement, and Bluetooth transmits data, eliminating the wires and bulky amplification unit. This is just one example of how previous sleep tracking technologies can be modernized and miniaturized using common tools in HCI. While effective for sleep tracking, the use of additional sensors could enable recording of physiological information related to dream content, such as emotional expression.

#### 2.7.2. Fascia

**Fig. 4.**
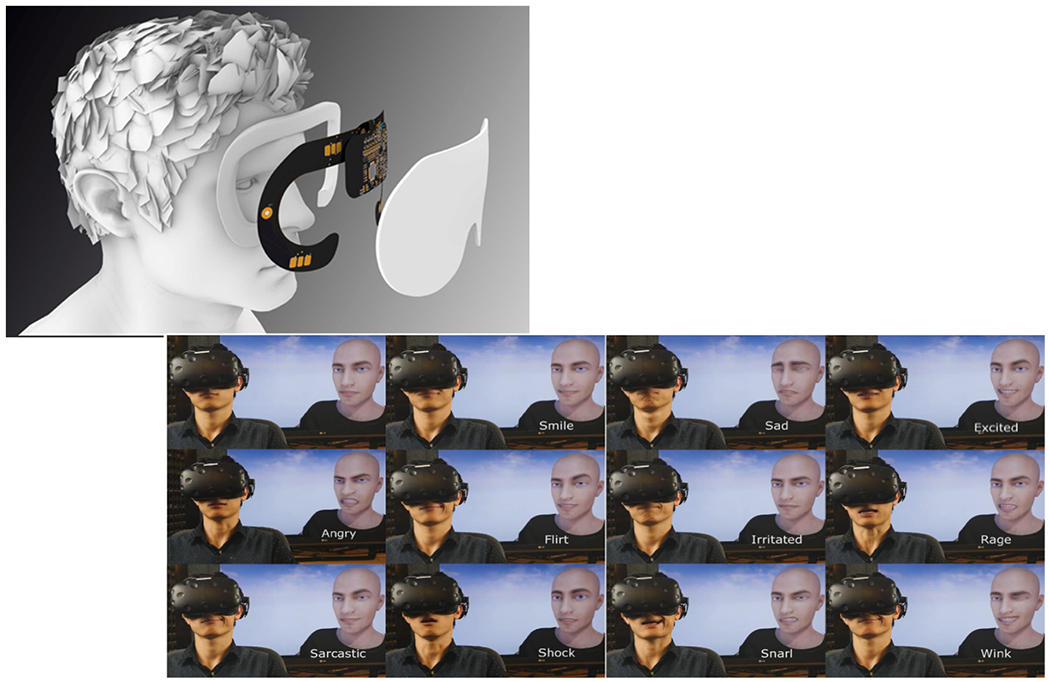
(top) The Fascia device, a smart sleep mask that collects EEG, EOG, EMG, heart rate, head movement and skin temperature. (bottom) The user’s real-time expression and emotion are mapped into the user’s VR avatar.

To further improve on the recording of multiple physiological signals, we developed *Fascia*, a smart sleep mask that simultaneously collects EEG, EOG, EMG, heart rate, head movement and skin temperature. This device is an update of a similar device built to collect physiological data from within a VR facemask, the *PhysioHMD* (Bernal, Yang, Jain, & Maes, 2018). The Fascia device is a flexible circuit in the shape of a sleep mask (Fig. 4), which aims to gather all relevant PSG data without disturbing sleep quality. The device integrates the sensors close to the skin, while two printed circuit boards house the components for signal processing and storage farther away from the skin. The prototype is designed to maximize the quantity and quality of sensor signals, as well as user comfort, to produce accurate data and reduce the first night effect (Agnew, Webb, & Williams, 1966). The prototype can also detect emotional expression via facial EMG and we previously demonstrated a system for recording the emotion of a VR user to an avatar, by mapping the user’s facial expression during a VR experience to a 3d rigged model avatar. This presents a possibility for recording emotional expressions and displaying them on a dream avatar, since prior research has demonstrated that frowning and smiling muscle tension during sleep corresponds with dreamed emotional content (Perlis, 1991; Rivera-García et al., 2019)

#### 2.7.3. Sleep scoring

There has been a significant increase in research devoted to machine learning algorithms for automatic sleep staging using wearable devices and even radio signals (Zhao, Yue, Katabi, Jaakkola, & Bianchi, 2017), or different types of EEG, such as stickers or in-ear EEG (Nakamura, Goverdovsky, Morrell, & Mandic, 2017; Stochholm, Mikkelsen, & Kidmose, 2016). However, most of these provide offline and not real-time sleep staging that is necessary for dream engineering techniques. Therefore, we developed a Deep Convolutional Neural Network that automatically computes sleep stages in real-time from single channel EEG. It has an overall accuracy of 83.5% for 5-stage classification of sleep stages ( + wake) on 4 test nights using power spectral analysis using the open Sleep-EDF dataset (Koushik et al., 2019). The algorithm processes the EEG data streamed via Bluetooth to a smartphone app. It scores in real-time sleep stages (30-second epochs) and does not require an offline analysis or server-client architecture as used in commercially available EEG headbands. The current algorithm has been tested with a customized EEG based on the commercially available *Muse* EEG. The app and algorithm are open-sourced and can be adapted to take in single-channel recordings from any wearable EEGs such as *Fascia.* We aim to use this algorithm for real-time interventions and integrated with other devices for sensory stimulation described throughout this paper.

### 2.8. Summary

We reviewed a wealth of devices from HCI designed and built to generate and maintain simulated virtual worlds, and provided examples of specific HCI devices that have been adapted to the sleep science field to experimentally modulate the sleeping body and track concurrent changes in dream content. In Table 2 we list existing dream engineering techniques and devices alongside ideas for future developments at the sensory interface of sleep, and describe possible functions of these technologies for improving sleep quality, enhancing memory, or generating specific dream content. Importantly, these devices could provide low-cost options to manipulate dreaming in the wild.

## 3. Future visions, ethical considerations and conclusions

### 3.1. Nighttime neuroprosthetics and BCI control

It was recently shown that controlling a Brain-Computer Interface (BCI) device is possible from within a lucid dream. Mallett (2019) showed that using a consumer BCI, a lucid dreamer was able to, while asleep, control a moving block on a computer screen as instructed. While preliminary, it is worth considering the application of BCIs to developments in dream engineering. For instance, the use of BCIs are increasingly common for neuroprosthetics, e.g. a BCI can detect motor commands (‘move arm’) from the sensorimotor cortex and translate this signal to a neuroprosthetic limb (Müller-Putz, Scherer, Pfurtscheller, & Rupp, 2005). BCI neuroprosthetics have even been used to restore walking in individuals with spinal cord injury (King, Wang, McCrimmon, Chou, Do, & Nenadic, 2014). However a major area of current research is to develop BCIs that are able to integrate more seamlessly with the body schema. Currently the use of BCI neuroprosthetics requires long periods of motor imagery practice, as individuals must re-learn how to send a motor command to control an external limb. VR has been used as a more immersive way to train individuals to control a virtual avatar using a BCI, prior to learning to control a real neuroprosthetic (De Mauro et al., 2010). Given the benefits of motor imagery and VR training for BCIs, a future possibility may be to use lucid dreaming to practice controlling a BCI while also exploring the use of the dreaming body in the dreamworld. Blumberg and Dooley (2017) have likewise suggested that adaptation to neuroprosthetics may benefit from nighttime stimulation similar to twitching, in order to fully integrate the sensory feedback of a neuroprosthetic limb into the body schema, essentially completing the dream engineering circuitry from bottom-up to top-down.

While considering such exciting prospects for the future for dream engineering, we also must consider the potential risks of developing dream engineering to such an extent. For instance, dreams could be directly recorded to a BCI avatar (which highlights the importance of consent), or technology could be directly controlled from within a dream (which highlights the role of agency of a dreamer acting in the real world). These are important ethical considerations that parallel others in the field of neuroengineering, which we discuss further below.

### 3.2. Ethical considerations

The ethics of dream engineering and sleep manipulation is a critical public discussion to address as experimental approaches and commercial products are increasingly developed. Perhaps of unique importance to the future of dream engineering is a consideration of the vulnerable state of a sleeping person, and the intimate relationship between a person and their dreams. Experimental approaches to influencing dreams in clinical settings, at home or in a sleep laboratory must consider potential threats to avoid the misuse of these technologies, prevent harmful sleep interventions and protect the safety of an individual.

Can dream engineering be harmful? Concepts of mind-control and inception, introducing ideas into an individual’s memory without their consent or even conscious awareness, harken back to public concerns around subliminal persuasion. At present, most dream engineering applications require priming in wakefulness, i.e. within an individual’s awareness, in order to influence dreams or reactivate memories during sleep (see Table 1). However, evidence is now emerging that sleep-learning, i.e. forming new memories while asleep, is possible. For instance, Arzi et al. (2014) paired the scent of cigarettes with that of rotten fish to participants who were smokers; presenting these scent-pairs during sleep alone (but not during wake) led to a reduction in cigarette smoking, meaning the negative association was learned during sleep and influenced subsequent behavior. While this is a positive outcome, there are real concerns that manipulating dreams or sleep-learning can have negative outcomes. These range from simple possibilities of inducing nightmares via unpleasant sensory stimulation, to more complex possibilities of selectively enhancing or weakening implicit associations – creating political bias or sexual attraction, among others.

At a more basic level, even dream engineering that is beneficial for an individual may be at the expense of other natural dreaming processes. For instance, if dream engineering is used on a nightly basis to trigger pleasant rose-scented dreams, does it interfere with the natural process of emotion regulation, the ‘overnight therapy’ of normal dreaming? If targeting learning a new language, could other declarative memories weaken? Thus, a critical avenue of research is evaluating the extent to which repeated or prolonged application of stimulation techniques can interfere with natural sleep function. Similar cautions have recently been raised in the field of lucid dreaming. Despite the numerous benefits afforded by lucid dreams, researchers argue that repeatedly practicing techniques of lucid dream induction, such as questioning reality during the day (termed reality checks) or disrupting the normal sleep pattern could have detrimental effects on psychology (Soffer-Dudek, 2020).

Finally, the state of immobility and absence of voluntary control make a sleeping person particularly vulnerable to external influences. Indeed, dream engineering approaches are designed to be nearly imperceptible in order to not waken an individual, and in experimental studies participants are unaware of whether they have been exposed to stimulation during sleep or not. Thus, individuals by design are unaware of and have no control over the application of dream engineering as it is occurring. It is necessary to continue to evaluate the potential misuses of dream engineering technology, including purposeful misuses for personal or political gain, but also unintentional misuses that interfere with natural sleep emotion or memory processing functions. In parallel with these ethical considerations, we suggest that a minimalist approach to dream engineering using simple sense stimulation techniques, technologies, and targets carries promise for numerous positive outcomes, and we encourage future research that keeps ethical responsibility at the forefront of dream engineering development.

### 3.3. Conclusions

In this paper, we offer a vision for the application of a wide range of sensory stimulation technologies to the area of sleep and dream engineering. We begin by emphasizing the causal role of the body in dream generation, outlining bodily sensations that serve as a bottom-up source of dreaming and identifying isomorphisms between physiology of the sleeping body and phenomenology of the dream. We further outline top-down processes which shape dream content: that memories are used as material for creation of dream content, with current emotion as a driving force behind associative connections, and generative sense making as an updating process to incorporate current sensations. Accordingly, we justify our approach of dream engineering via bodily stimulation, moving beyond neurocognitive *brain in a vat* models of dreaming. The dream is understood as in circuit with the body. We then identify past protocols for influencing dream content, many of which have taken advantage of dreaming in circuitry with the body, i.e. linking thirst to dreams of water. Considering other areas that might afford tools for engineering sensory content in simulated worlds, we turn to Virtual Reality. We elucidate parallels between dreaming as a simulation of waking experiences and VR as a simulated world and we describe the development of new VR technologies, such as haptic and olfactory stimulation devices designed to simulate waking sensations and engineer plausible world simulations. We propose that an understanding of these Human Computer Interaction technologies, in the context of sleep and dream research, will enable developments in dream engineering. We outline a collection of relevant VR technologies from the HCI field, categorizing them by sense, including those which potentiate auditory, olfactory, temperature and haptic stimulation. We hope these technologies, which have been engineered for high mobility and low cost, can be transferred directly to the field of dream engineering. We also describe relevant tools created to interface with sleep and dreams, including sleep tracking devices that offer clear links for integration with VR technologies. We close by discussing possible future directions in sleep engineering, dream direction and nighttime neuroprosthetics, and the ethics of a world in which targeted dream engineering and sleep manipulation are feasible.

## Figures and Tables

**Table 1:** Overview of Stimulation Techniques. Pre-sleep priming presents a stimulus before sleep, e.g., a video or music to influence dream content in subsequent sleep. Dream incubation involves pre-sleep rehearsal of content, such as visualization of a rescripted nightmare, repeating an intention to become lucid (lucid dream incubation), or focusing on a personal problem to incubate a creative solution. Dream direction applies a sensory stimulus during sleep and relies on implicit associations to sensation to direct dreams: a pleasant scent to ameliorate dream emotion, muscle stimulation to augment dream movement, speech to direct dream narrative. Of note, pre-sleep priming, dream incubation, and dream direction may affect dream content in relatively metaphorical or idiosyncratic, but nonetheless functional ways. Rhythmic entrainment acts on physiological rhythms during sleep, from fast rhythms like neural oscillations to slower rhythms like respiration or circadian changes in temperature; while entrainment does not necessarily influence dream content, it may improve sleep functions. Finally, in targeted reactivation, a stimulus is paired with specific content during wake, and when the stimulus is re-presented during sleep its associated content is reactivated. Targeted reactivation can enhance consolidation of specific memory traces (targeted memory reactivation), trigger imagery that was rehearsed prior to sleep (targeted dream reactivation), or induce lucidity (targeted lucidity reactivation).

	Wake		Sleep	
	Stimulus	Content	Stimulus	Content
Pre-Sleep Priming	✔	X	X	✔
Dream Incubation	X	✔	X	✔
Dream Direction	X	X	✔	✔
Rhythmic Entrainment	X	X	✔	X
Targeted Reactivation	✔	✔	✔	✔

**Table 2:** Dream Engineering Techniques, Technologies and Functional Targets.

Sense	Technique	Technology	Function
Sound	Targeted Reactivation^1^ (TMR, TDR, TLR), Dream Direction (DD), Rhythmic Entrainment^1^ (RE)	Bone conduction, Dormio, Bluetooth speaker	Improving sleep depth and sleep efficiency (RE), alleviating nightmares (TDR, TLR), augmenting creativity (TDR), ameliorating dream valence (TLR, DD), enhancing memory (TMR)
Visual	Targeted Reactivation (TLR), Pre-Sleep Priming (PSP), Rhythmic Entrainment (RE)	Light stimulation masks (e.g. DreamLight), Head-Mounted Displays, video monitors	Augmenting creativity (TLR), ameliorating dream valence (TLR, PSP), alleviating nightmares (TLR, PSP), improving sleep depth and efficiency (RE)
Temperature	Dream Direction (DD), Rhythmic Entrainment (RE)	ChilliPad, thermal sheet, heated eyemasks, hand warmers	Ameliorating dream valence (DD), alleviating nightmares (DD), reducing sleep onset latency (RE), improving sleep depth and efficiency (RE)
Haptic/Proprioceptive	Dream Direction (DD), Rhythmic Entrainment (RE)	Pressure cuff, Electrical Muscle Stimulation, Galvanic Vestibular Stimulation, rocking	Ameliorating dream valence (DD), reducing sleep onset latency (RE), motor learning (DD)
Smell	Targeted Reactivation (TMR, TDR), Rhythmic Entrainment (RE), Dream Direction (DD)	Essence, olfactometer, scent diffuser	Enhancing memory (TMR), improving sleep depth and efficiency (TMR, RE), alleviating nightmares (TDR, DD), reducing sleep onset latency (RE), ameliorating dream valence (DD)

1 Targeted Reactivation includes Targeted Memory Reactivation (TMR), Targeted Dream Reactivation (TDR) and Targeted Lucidity Reactivation (TLR). Rhythmic Entrainment (RE) can act on neural oscillations, respiratory, or circadian rhythms among others. Pre-Sleep Priming (PSP) presents a stimulus prior to sleep intending to influence subsequent dreams, and Dream Direction (DD) applies a stimulus only during sleep to influence dreams.
